# Novel Associations for Hypothyroidism Include Known Autoimmune Risk Loci

**DOI:** 10.1371/journal.pone.0034442

**Published:** 2012-04-06

**Authors:** Nicholas Eriksson, Joyce Y. Tung, Amy K. Kiefer, David A. Hinds, Uta Francke, Joanna L. Mountain, Chuong B. Do

**Affiliations:** 23andMe, Inc., Mountain View, California, United States of America; University of Oklahoma and Oklahoma Medical Research Foundation, United States of America

## Abstract

Hypothyroidism is the most common thyroid disorder, affecting about 5% of the general population. Here we present the current largest genome-wide association study of hypothyroidism, in 3,736 cases and 35,546 controls. Hypothyroidism was assessed via web-based questionnaires. We identify five genome-wide significant associations, three of which are well known to be involved in a large spectrum of autoimmune diseases: rs6679677 near *PTPN22*, rs3184504 in *SH2B3*, and rs2517532 in the HLA class I region (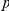-values 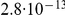, 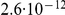, and 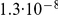, respectively). We also report associations with rs4915077 near *VAV3* (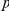-value 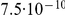) and rs925489 near *FOXE1* (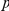-value 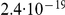). *VAV3* is involved in immune function, and *FOXE1* and *PTPN22* have previously been associated with hypothyroidism. Although the HLA class I region and *SH2B3* have previously been linked with a number of autoimmune diseases, this is the first report of their association with thyroid disease. The *VAV3* association is also novel. We also show suggestive evidence of association for hypothyroidism with a SNP in the HLA class II region (independent of the other HLA association) as well as SNPs in *CAPZB*, *PDE8B*, and *CTLA4*. *CAPZB* and *PDE8B* have been linked to TSH levels and *CTLA4* to a variety of autoimmune diseases. These results suggest heterogeneity in the genetic etiology of hypothyroidism, implicating genes involved in both autoimmune disorders and thyroid function. Using a genetic risk profile score based on the top association from each of the five genome-wide significant regions in our study, the relative risk between the highest and lowest deciles of genetic risk is 2.0.

## Introduction

Hypothyroidism is characterized by deficiencies of thyroid hormones T3 (triiodothyronine) and T4 (thyroxine). Thyroid hormones are primarily responsible for the regulation of metabolism, but also play a major role in development. Hypothyroidism is typically marked by high thyroid-stimulating hormone (TSH) levels (usually indicative of impaired thyroid function). However, this is not always the case. For example, reduced T3/T4 levels may be caused by insufficient generation of TSH by the pituitary gland rather than thyroid dysfunction. While iodine deficiency is the most common cause of hypothyroidism worldwide, and there are rare forms of congenital hypothyroidism with a number of genetic causes [1], most cases in the developed world are due to autoimmune hypothyroidism (e.g., Hashimoto or Ord thyroiditis). Over the last five years, hundreds of genetic variants have been found that predispose to various autoimmune diseases [2], with many shared across multiple autoimmune diseases [3].

There has been only one published genome-wide association study (GWAS) of hypothyroidism, carried out in a sample of 1317 hypothyroidism cases and 5053 controls determined algorithmically from five electronic medical record databases. They found one association, near *FOXE1* (forkhead box E1), also known as *TTF-2* (thyroid transcription factor 2) [4]. Candidate gene studies have also suggested links between autoimmune hypothyroidism and *PTPN22* (protein tyrosine phosphatase, non-receptor type 22 (lymphoid)), as well as the HLA (human leukocyte antigen) class II region, *CTLA4* (cytotoxic T lymphocyte antigen 4), and 8q23-24 [5]–[13]. Graves' disease (another autoimmune thyroid disease, characterized by hyperthyroidism) has been studied in several GWAS, with many loci discovered [14]–[17].

In addition to hypothyroidism, *FOXE1* has previously been associated with thyroid cancer and TSH levels [18]. A second SNP near this gene (rs755109) has also been associated with TSH levels in an isolated Pacific Island population [19]. *FOXE1* is also involved in thyroid development and coding mutations cause congential hypothyroidism [20]. As untreated individuals with hypothyroidism typically have high TSH levels, it is possible that a GWAS of TSH levels could detect loci involved in hypothyroidism. These facts raise the question of whether other variants associated with thyroid function also influence hypothyroidism risk.

The genetic determinants of TSH levels are partially understood, with several established associations from GWAS, including *PDE8B* (phosphodiesterase 8B) [19], [21] *CAPZB* (capping protein (actin filament) muscle Z-line, beta) [22], and *NKX2-1* (NK2 homeobox 1), also known as *TTF-1* (thyroid transcription factor 1) [18]. *CAPZB* has also been associated with thyroid volume [23]. These genes have strong links to thyroid function: in addition to the thyroid transcription factors, *PDE8B* is primarily expressed in the thyroid [24] and encodes a phosphodiesterase with a high affinity for cAMP, which mediates TSH effects in the thyroid [25].

In this paper, we report on the largest GWAS to date of hypothyroidism. We find five variants significantly associated with hypothyroidism. Two are non-synonymous variants in genes associated with many autoimmune diseases (*PTPN22* R620W and *SH2B3* (SH2B adaptor protein 3) R262W) and a third is in the HLA class I region. A fourth is found in an intron of *VAV3* (vav 3 guanine nucleotide exchange factor), a gene plausibly involved in immune function. The final variant is located upstream of the thyroid transcription factor *FOXE1*. In addition, among the SNPs marginally associated with hypothyroidism, we observe associations with two genes that have been linked to TSH levels: *PDE8B* and *CAPZB*. We also replicate a previously reported association of *CTLA4* with hypothyroidism [13].

## Results

We performed a GWAS in 3,736 cases and 35,546 controls from the customer base of 23andMe, Inc., a personal genetics company. All participants were of primarily European ancestry and were at most distantly related to each other. Figure S1 shows further details about the population structure. Hypothyroidism was assessed using online self-report. Briefly, participants responded to questions about their hypothyroidism diagnosis and related thyroid issues using web-based surveys. We classified as cases individuals who had been diagnosed with hypothyroidism, had elevated TSH levels, or were taking thyroid hormone replacement medication. Controls reported no to at least one of the above questions and yes to none of them. Participants reporting hyperthyroidism, thyroid cancer, thyroid removal, or treatment with radioactive iodine for hyperthyroidism were excluded from both the cases and controls. Details about the cohort can be found in Table 1 and the Methods. All analyses were controlled for age, sex, and five principal components. In addition, a conditional analysis was performed, adding the five genome-wide significant SNPs as covariates. Manhattan and quantile-quantile plots for both analyses are provided in Figures S2 and S3.

**Table 1:** Cohort statistics.

	Number	Male	Female	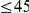	46–55	56–65	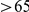	V1	V2	V3
Control	35546	22446	13100	18941	5528	6142	4935	271	15699	19572
Case	3736	953	2783	979	640	1120	997	29	1694	2013

Participants broken down by sex, age, and genotyping platform. V1, V2, and V3 refer to the three platforms used in this study, see Methods.

Table 2 shows lead SNPs from loci with 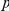-values under 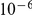 for hypothyroidism. Under our threshold for genome-wide significance of 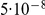, five regions are significant (*FOXE1*, *PTPN22*, *SH2B3*, *VAV3*, and the HLA region. The strongest association is with rs925489, with a 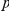-value of 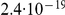 and odds ratio (OR) of 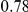, near *FOXE1* (Figure S4). This variant has been associated with hypothyroidism [4]. It is in linkage disequilibrium (LD) with rs965513, which has been associated with thyroid cancer and TSH levels [18] and is in weak LD (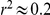) with rs1867277, which has been associated with thyroid cancer and shown to affect *FOXE1* transcription [26]. Homozygous loss-of-function mutations in this gene cause congenital hypothyroidism due to thyroid dysgenesis and other developmental abnormalities [20].

**Table 2:** SNPs associated with hypothyroidism at 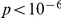.

SNP	Chr.	Pos.	Region	Alleles	MAF	HWE	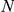	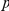 -value	OR
rs925489	9	99586421	*FOXE1*	T/C	0.332	0.69	38947	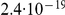	0.78 (0.74–0.82)
rs6679677	1	114105331	*PTPN22*	C/A	0.091	0.34	38959	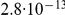	1.36 (1.26–1.48)
rs2476601	1	114179091	*PTPN22*	G/A	0.092	0.26	39256	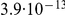	1.36 (1.25–1.47)
rs3184504	12	110368991	*SH2B3*	T/C	0.497	0.49	39245	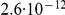	0.84 (0.79–0.88)
rs4915077	1	108167539	*VAV3*	T/C	0.084	0.76	39248	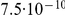	1.30 (1.20–1.42)
rs2517532	6	31126386	HLA	G/A	0.403	0.094	39225	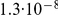	0.86 (0.82–0.91)
rs2516049	6	32678378	HLA	T/C	0.307	0.13	39241	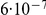	1.15 (1.09–1.21)

All genomic positions are given with respect to NCBI build 36.3. Alleles are listed as major/minor and are specified for the forward strand. 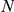 refers to the number of people successfully genotyped for each SNP. Odds ratios are per copy of the minor allele. One SNP is listed per region of the genome with the exception of HLA, which shows evidence of two independent signals, and *PTPN22*, for which we have included rs2476601, the non-synonymous change R620W.

The second association is with rs6679677, with a 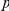-value of 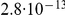 and OR of 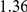 near *PTPN22* (Figure S5). The SNP rs6679677 is in LD (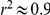) with rs2476601 (the missense mutation R620W in *PTPN22*). This mutation has previously been associated with Hashimoto thyroiditis in a relatively small candidate gene study [5]. *PTPN22* also has well-established associations with multiple autoimmune conditions [27], including type 1 diabetes, rheumatoid arthritis, systemic lupus erythematosus, juvenile idiopathic arthritis [28], Graves' disease [29], [30], systemic sclerosis [31], and alopecia areata [32].

Next, we see a novel association with rs3184504, a missense mutation (R262W) in *SH2B3*, with a 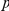-value of 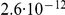 and an OR of 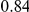 (Figure S6). This SNP has not previously been associated with thyroid disease; however, it has been associated with a number of autoimmune diseases, including type 1 diabetes [33], celiac disease [34], rheumatoid arthritis [35], and multiple sclerosis [36], as well as with hypertension and myocardial infarction [37]. The T allele of rs3184504 is the variant associated with increased risk in our data and corresponds to the W allele in the protein, which is also the risk variant for type 1 diabetes and rheumatoid arthritis and the protective variant for celiac and multiple sclerosis.

**Figure 1:**
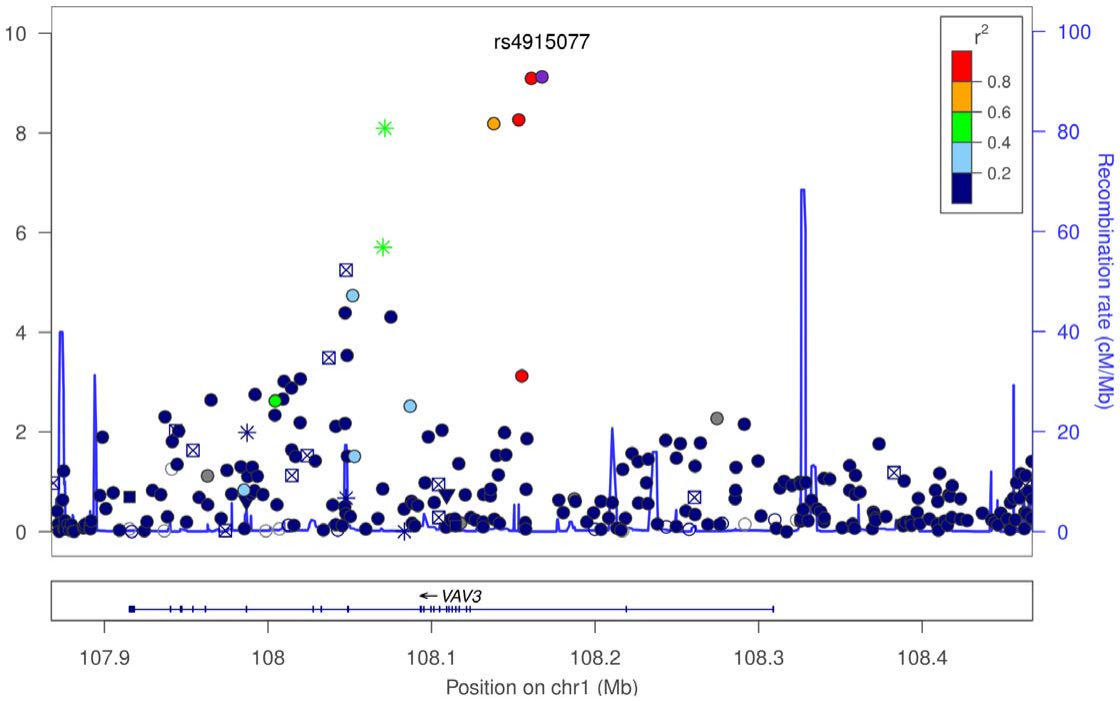
SNPs in the *VAV3* region. In the plot, circles represent unannotated SNPs, upside-down triangles represent non-synonymous variants, and boxes with an “x” are SNPs in regions that are highly conserved across 44 placental mammals. Colors depict the squared correlation (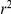) of each SNP with the most associated SNP (i.e., rs4915077, shown in purple). Gray indicates SNPs for which 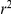 information was missing. Plots were produced using the LocusZoom program [50].

The next association, which is novel, is with rs4915077, in an intron of *VAV3*. This SNP has a 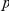-value of 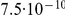 and OR of 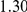. See Figure 1 for SNPs in this region.

**Figure 2:**
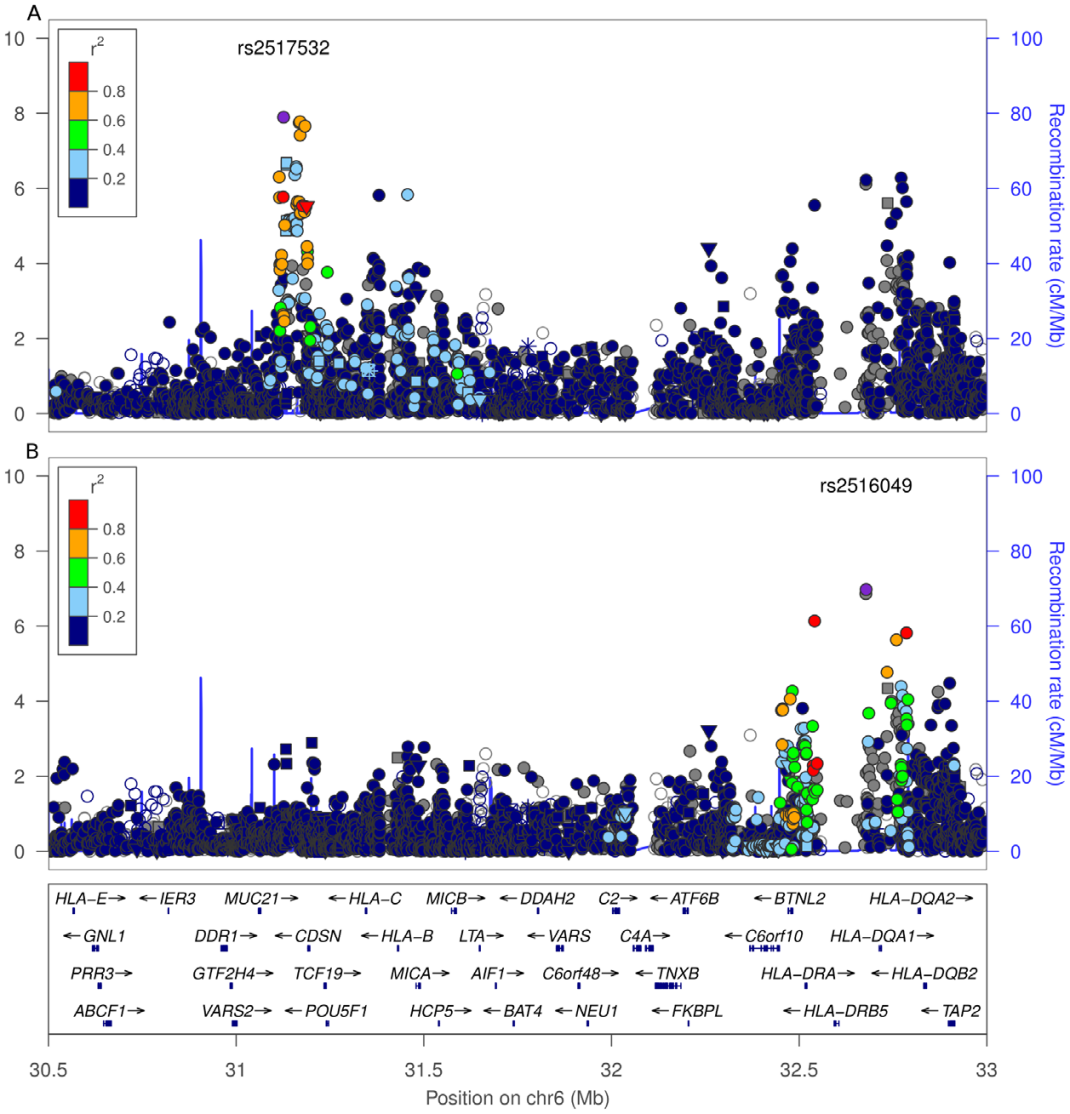
SNPs in the *HLA* region. (A) shows statistics for the main GWAS, (B) shows statistics conditioned on 5 genome-wide significant SNPs (rs925489, rs6679677, rs3184504, rs4915077, rs2517532). For details, see Figure 1.

Finally, we found two associations between the HLA region and hypothyroidism (Table 2). The first, rs2517532, is genome-wide significant with a 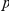-value of 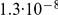 and OR of 0.86. It lies in the HLA class I region, between *HLA-E* and *HLA-C*. The second association (rs2516049, about 2 mb away in the HLA class II region, near *HLA-DRB1*) shows only suggestive evidence of association (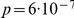). However, a conditional genome-wide analysis (conditioning on the five significant associations) reveals that this second HLA association is independent of the first (see Figure 2).

Using the five genome-wide significant SNPs, we constructed risk scores for all participants. In the top decile of risk, there were 555 cases and 3373 controls versus 277 cases and 3650 controls in the bottom decile, giving a relative risk of approximately 2.0.

Table 3 shows three associations for hypothyroidism with 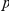-values under 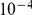 that are relevant for other conditions. Near *CAPZB* (Figure S7), rs1472565 is in moderate LD (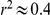) with rs12091047 (associated with thyroid volume [23]) but not in LD with rs10917469 (associated with TSH levels [22]). The SNP we observe near *PDE8B*, rs4704397 (Figure S8), has also been associated with TSH levels [21]. Finally, rs231779 near *CTLA4* (Figure S9) has been associated with many other autoimmune diseases as well as with autoimmune hypothyroidism in candidate gene studies [3], [8]. All SNPs with 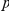-values under 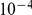 are shown in Table S1.

**Table 3:** Selected SNPs suggestively associated with hypothyroidism.

SNP	Chr.	Pos.	Region	Alleles	MAF	HWE	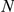	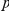 -value	OR
rs1472565	1	19,627,617	*CAPZB*	T/C	0.478	0.39	39249	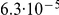	1.107 (1.05–1.16)
rs4704397	5	76,554,198	*PDE8B*	G/A	0.390	0.98	21622	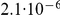	1.179 (1.10–1.26)
rs231779	2	204,442,732	*CTLA4*	C/T	0.366	0.81	39254	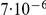	1.126 (1.07–1.19)

Three SNPs with connections to other conditions that are suggestively associated with hypothyroidism. *CAPZB* and *PDE8B* have been associated with TSH levels and *CTLA4* with a variety of autoimmune diseases, including autoimmune hypothyroidism. See Table 2 for nomenclature, Table S1 for all SNPs with 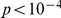 and Table S2 for 107 SNPs involved in autoimmune disease.

To search for further SNPs shared with other autoimmune diseases, we investigated a list of 107 SNPs that were studied across 7 autoimmune diseases [3]. Among this list, only the *CTLA4*, *PTPN22*, and *SH2B3* loci show significant association with hypothyroidism after correction for 107 multiple tests. See Table S2 for details for all 107 SNPs.

## Discussion

**Figure 3:**
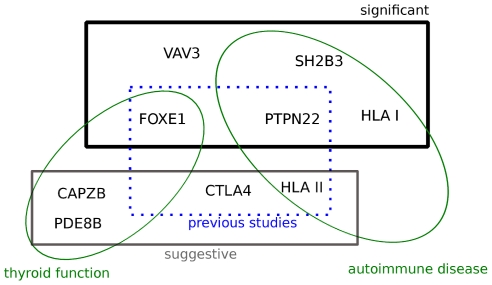
Summary of results. Regions associated with hypothyroidism classified by signficance level (genome-wide or suggestive), known function (thyroid versus autoimmune), and whether they had previously been associated with hypothyroidism.

We have found five genome-wide significant associations for hypothryoidism (near *FOXE1*, *PTPN22*, *SH2B3*, *VAV3*, and the HLA class I region) as well as four others with suggestive evidence of biological interest (near *CAPZB*, *PDE8B*, and *CTLA4* as well as in the HLA class II region). Of these, six seem to be involved in immune function (*PTPN22*, *SH2B3*, *VAV3*, *CTLA4*, and the two in HLA) and three in thyroid function (*FOXE1*, *CAPZB*, and *PDE8B*); see Figure 3. This suggests that while autoimmune loci may play a predominant role in genetic predisposition for hypothyroidism, there may be smaller effects due to genes more directly related to thyroid function and hormone levels.

Three of these associations are novel: the non-synonymous change R262W in *SH2B3*, the SNP rs4915077 near *VAV3*, and rs2517532 in the HLA class I region. The *SH2B3* and HLA associations reinforce the connection between hypothyroidism and autoimmune genetics. *VAV3* is a guanine nucleotide exchange factor for Rho guanosine triphosphatases. It has not yet been associated with autoimmune disease or thyroid function. However, it has been proposed as the candidate gene in the Idd18.1 region linked with type 1 diabetes in mouse [38], and the Vav1/Vav2/Vav3 family is necessary for adaptive immune function in mouse [39]. *VAV3* is also expressed in the thyroid [40] and is down-regulated in a subset of thyroid tumors [41]. The second, suggestive association in the HLA class II region, rs2516049, lies near *HLA-DRB1*. Non-synonymous variants in *HLA-DRB1* have been associated with autoimmune thyroiditis in humans and in mice [6].

We have also replicated several previously discovered associations. *FOXE1* has recently been associated with hypothyroidism (as well as several other thyroid conditions) in a GWAS with 1,317 hypothyroidism cases determined from medical records [4]. Their strongest association was at rs7850258, with a reported OR of 0.74 (95% confidence interval of 0.67–0.82). For this SNP, we see a very similar OR of 0.78 (0.74–0.82). We have found one more association with a variant well known to be involved in autoimmune disease: R620W in *PTPN22*. This association was also seen in Denny et al. [4] (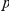-value of 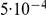, OR of 1.29) as well as in a small candidate gene study of Hashimoto thyroiditis [5] (OR of 1.77 (1.31–2.40) in a sample of 194 cases and 2064 controls). We observe an OR for this SNP of 1.36 (1.25–1.47), which is similar to previous studies, despite slightly different phenotypes.

While many autoimmune diseases share genetic risk factors [42], there is evidence that these diseases form separate clusters based on genetics [3], [43]. For example, the 620W allele of *PTPN22* has a protective effect in Crohn's disease [44] but is the risk allele for type 1 diabetes and others [45] (and hypothyroidism as reported here). *SH2B3* also shows opposite directions of effect in multiple sclerosis and celiac compared to rheumatoid arthritis, psoriasis, type 1 diabetes, and hypothyroidism [3]. Thus our results begin to place hypothyroidism on the spectrum of autoimmune disease.

Our web-based design leads to a number of interesting trade-offs. On the one hand, the ease of online phenotype collection allows us to cheaply amass a very large study population (e.g., 3,736 cases in our study, compared to 1,317 cases in the only previous GWAS of hypothyroidism [4]). On the other hand, due to the nature of online self-reported data, we did not gather clinical measures such as TSH levels or hypothyroidism symptoms. The consequences of potential case misclassification arising from the unavailability of clinical data may manifest in two different ways. If some of our reported cases were misclassified at random (i.e., if a fraction 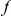 of the cases were actually controls who randomly reported having hypothyroidism), then the impact on our study would be a decrease in power as a consequence of a downward bias in the odds ratios observed in our study population, with no increase in false positive rate. In our study, however, the compensatory gain in power from having a larger sample more than makes up for the reduction in power due to random misclassification. For instance, conservatively assuming an 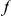 as high as 0.2, our power to detect the HLA class I association in Table 2 was 27%; reducing the sample size in half in order to obtain 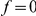 results in a power reduction to 15%. In practice, the close similarity of the odds ratios reported here with previous estimates suggest that random misclassification is unlikely to substantially affect our results.

A separate concern is whether a substantial proportion of the cases in this study were not randomly misclassified controls but rather individuals who actually exhibited symptoms for a separate health condition (e.g., elevated TSH levels unrelated to hypothyroidism). Our study does not exclude this possibility. For example, about 7.5% of females and 2.8% of males are estimated to have elevated TSH levels and 2–3% of individuals are estimated to have overt symptoms of hypothyroidism [45]. These estimated rates of hypothyroidism are lower than the 9.5% prevalence of hypothyroidism (defined as either elevated TSH levels or hypothyroidism diagnosis) in our cohort, although the hypothyroidism is generally thought to be under-diagnosed (or possibly over-diagnosed in a health-conscious group of participants). As many of the SNPs discussed here show pleiotropic effects across autoimmune diseases or thyroid conditions, and as hypothyroidism is difficult to exactly define in a retrospective, web-based design, ultimately, the loci discovered here should be further replicated in a more deeply phenotyped hypothyroidism population, to determine the interplay between thyroid function genes and immune system genes that leads to this disorder.

## Methods

### Cohort

All participants in the study were customers of 23andMe, Inc., a personal genetics company, who had been genotyped as part of the 23andMe Personal Genome Service®. Individuals included in the cohort were selected for being of primarily European ancestry, as determined through an analysis of local ancestry via comparison to the three HapMap 2 populations, using an unpublished method substantially similar to Falush et al. [46]. All participants had over 97% of their genome estimated to be most similar to the HapMap CEU population. This subset was selected as it is the largest relatively unstructured set within the 23andMe customer base.

A maximal set of unrelated individuals was chosen for the analysis using a segmental identity-by-descent (IBD) estimation algorithm (as used in [47]). Individuals were defined as related if they shared more than 700 cM IBD, including both regions where the two individuals share either one or both genomic segments identical-by-descent. This level of relatedness (roughly 20% of the genome) corresponds approximately to the minimal expected sharing between first-cousins in an outbred population.

This study was conducted according to the principles expressed in the Declaration of Helsinki. The study protocol and informed consent form were approved by the external IRB, Ethical and Independent Review Services (E&I Review), which is accredited by the Association for the Accreditation of Human Research Protection Programs (AAHRPP). Informed consent was obtained from subjects online, in an IRB approved process. Our consent and privacy statement preclude the sharing of individual-level data without explicit consent. We have, however, shared summary statistics for all SNPs with 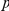-values under 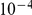 in Table S1.

### Genotyping

For the 23andMe cohort, DNA extraction and genotyping were performed on saliva samples by National Genetics Institute (NGI), a CLIA-certified clinical laboratory and subsidiary of Laboratory Corporation of America. Samples were genotyped on one of three different platforms. About half of the participants were genotyped on one of two platforms based on the Illumina HumanHap550+ BeadChip (called V1 and V2 in Table 1), which included SNPs from the standard HumanHap550 panel augmented with a custom set of approximately 25,000 SNPs selected by 23andMe. Two slightly different versions of this platform were used, as described in [47]. The remaining participants were genotyped on the Illumina OmniExpress+ Bead Chip. This platform has a base set of 730,000 SNPs. It was augmented by approximately 250,000 SNPs to make it approximately a superset of the HumanHap550+, as well as a custom set of about 30,000 SNPs. This platform was called V3 in Table 1. With the exception of the SNP rs4704397 near *PDE8B* (a V3-only SNP), all SNPs discussed here appeared at least on the V2 and V3 platforms that were used to genotype the vast majority of samples. Every sample that failed to reach a 98.5% call rate for SNPs on the standard platforms was re-analyzed. Individuals whose analyses failed repeatedly were re-contacted by 23andMe customer service to provide additional samples, as is done for all 23andMe customers. Our quality control procedures include genotyping the HapMap sample on our platforms and discarding discordant SNPs, manual examination of thousands of cluster plots, filtering based on Mendelian discordance in thousands of genotyped trios, filtering by Hardy-Weinberg, as well as independent validation of many probes. Quality control procedures for the genotyping are described in more detail in [47].

SNPs with a call rate under 95% or minor allele frequency under 0.01 were excluded from analysis. Call rates were calculated on a per-platform basis. Additionally, SNPs with Hardy-Weinberg 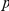-values [48] less than than 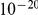 were excluded. Altogether, 870,065 SNPs (on the union of the two platforms) were retained with an average call rate of 99.78%.

### Phenotyping

Participants were able to fill out web-based questionnaires whenever they logged into their 23andMe accounts. Participants answered some of the following questions:

- Have you ever been diagnosed by a doctor with any of the following thyroid conditions? (asked as part of a medical history questionnaire)
  Hyperthyroidism
  Hypothyroidism
- Have you been diagnosed with any of the following? (asked as part of a questionnaire on baldness)
  Hyperthyroidism (overactive thyroid)
  Hypothyroidism (underactive thyroid)
- Have you ever been diagnosed with hypothyroidism (underactive thyroid)?
- Do you currently take medication for hypothyroidism (low thyroid hormone levels)?
- Have you ever been told by a doctor that your thyroid stimulating hormone (TSH) levels were elevated, indicating hypothyroidism?
- Have you ever been diagnosed with thyroid cancer?
- Have you ever received radioactive iodine treatment for goiter or hyperthyroidism (overactive thyroid)?
- Have you ever had all or part of your thyroid surgically removed?
- Have you ever been diagnosed by a doctor with any of the following common cancers? (asked as part of a medical history questionnaire)
  Thyroid cancer

Cases answered yes to hypothyroidism or to elevated TSH levels or to taking medication for hypothyroidism. Controls answered no to at least one of the qualifying questions and yes to none of them. Individuals reporting hyperthyroidism or thyroid cancer or treatment with radioactive iodine or thyroid removal were excluded, as all of these could cause hypothyroidism or could signal Graves' disease.

As customers of 23andMe, all participants had the opportunity to view reports based on their genetic information on over 100 traits and diseases. Among these diseases were reports on thyroid cancer (covering the *FOXE1* and *NKX2-1* SNPs from [18]) and Hashimoto thyroiditis (covering *PTPN22*). The context in which a question is asked can influence responses, as we have seen in a very different context in a previous paper [47]. While it is unlikely that a predicted high or low risk for thyroid cancer would lead to misreport of hypothyroidism, we sought to rule out any such bias in this case. Indeed, for *PTPN22*, there was no evidence of a difference in ORs for rs2476601 and hypothyroidism for people viewing their results before or after answering survey questions (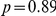 for interaction).

### Statistical analysis

For the association analysis, all 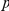-values were calculated using a likelihood ratio test for the logistic regression model, adjusting for sex, age, and projections onto the first five principal components of the genotype data matrix. The principal components used were calculated within the subset of 23andMe customers with primarily European ancestry, as described previously [49]. Figure S1 shows the first two principal components with self-reported ancestry overlayed for a subset of participants. The inflation factor without any principal components included in the model was 1.032. After including five, this decreased slightly to 1.029. See Figure S3 for the quantile-quantile plot. The phenotypic status of each individual was coded as 0 for unaffected individuals and 1 for affected individuals. Genotypes were coded 0, 1, or 2, to indicate the number of minor alleles present for the tested SNP (corresponding to a log-additive model of association). For the conditional analysis, the 5 SNPs found to be genome-wide significant were added as predictors to the model. For SNPs appearing on a subset of genotyping platforms, analyses were restricted to individuals who were typed. Reported odds ratios for each SNP relative to the minor allele were defined as the exponential of the regression coefficients, and the alleles used throughout refer to the plus strand of NCBI build 36.3 of the human genome. We used a cutoff for genome-wide significance of 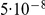 (corresponding to a Bonferroni correction assuming 1 million independent tests).

## Supporting Information

Figure S1 **Projections onto first two principal components for all participants in this study.** The country codes are based on self-reported ancestry from a subset of 3363 participants. These participants reported four grandparents born in the given country (or in the case of “AJ”, four grandparents of Ashkenazi Jewish ancestry). The label for a country is placed at the median position of all participants reporting such ancestry, and the size is proportional to the number of such reports. Note that the label size is not proportional to the actual density of each subgroup in the study (the densities are approximately 85% northern European and 5% each Ashkenazi, eastern European, and southern European).
(TIFF)

Figure S2
**Manhattan plots.** (A) Negative log 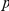-values for SNPs by genome position. Genome-wide significant SNPs are shown in red. (B) Same for conditional analysis adding 5 genome-wide signficiant SNPs (rs925489, rs6679677, rs3184504, rs4915077, rs2517532) as covariates.(TIFF)

Figure S3
**Quantile-quantile plot.** Observed 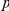-values versus theoretical 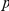-values under the null hypothesis of no association. The genomic control inflation factor for the study was 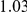 and is indicated by the red line. (A) Genome-wide analysis. (B) Conditional analysis with five SNPs included as covariates.(TIFF)

Figure S4
**SNPs in the** ***FOXE1*** **region.** In the plot, circles represent unannotated SNPs, upside-down triangles represent non-synonymous variants, and boxes with an “x” are SNPs in regions that are highly conserved across 44 placental mammals. Colors depict the squared correlation (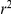) of each SNP with the most associated SNP (i.e., rs925489, shown in purple). Gray indicates SNPs for which 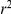 information was missing. Plots were produced using the LocusZoom program.(TIFF)

Figure S5 **SNPs in the** ***PTPN22*** **region.** For details, see Figure S4.
(TIFF)

Figure S6 **SNPs in the** ***SH2B3*** **region.** For details, see Figure S4.
(TIFF)

Figure S7 **SNPs in the** ***CAPZB*** **region.** For details, see Figure S4.
(TIFF)

Figure S8 **SNPs in the** ***PDE8B*** **region.** For details, see Figure S4.
(TIFF)

Figure S9 **SNPs in the** ***CTLA4*** **region.** For details, see Figure S4.
(TIFF)

Table S1
**SNPs with** 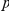 **-values under** 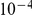 **.** All genomic positions are given with respect to NCBI build 36.3. Alleles are listed as major/minor and are specified for the forward strand. 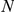 refers to the number of people successfully genotyped for each SNP. Odds ratios are per copy of the minor allele.(XLS)

Table S2
**SNPs identified in other autoimmune diseases.** The 107 SNPs studied across 7 autoimmune diseases in Cotsapas et al., 2011. Under a threshold of 
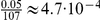
 for significance, only *PTPN22*, *SH2B3*, and *CTLA4* are significant. See Table S1 for details on columns.(XLS)
