# Supplementary figures and images for: Novel Associations for Hypothyroidism Include Known Autoimmune Risk Loci
Source: PLoS One. 2012 Apr 6;7(4):e34442. doi: 10.1371/journal.pone.0034442 (PMC3321023; doi:10.1371/journal.pone.0034442)

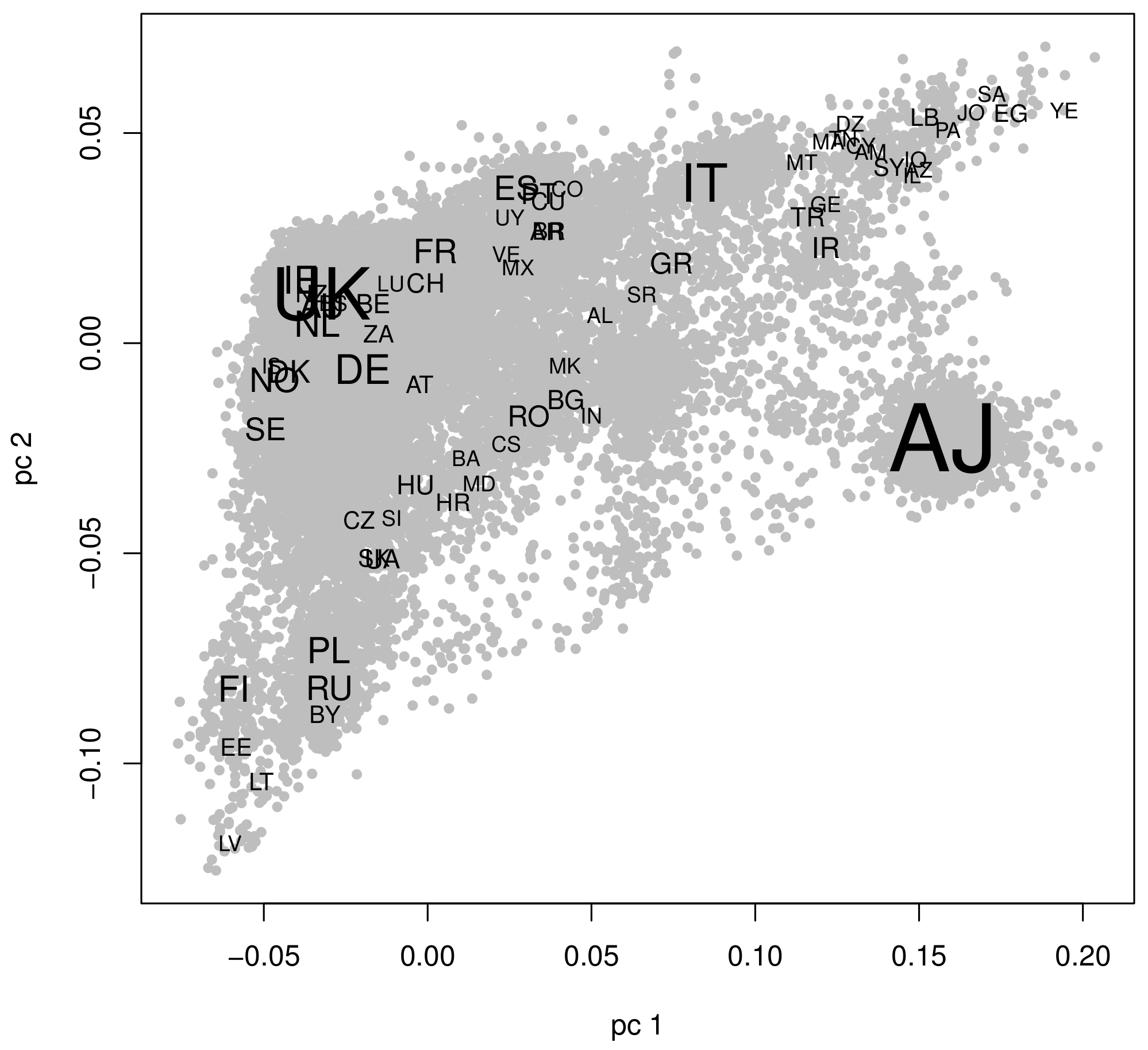

Supplement: Figure S1 — Projections onto first two principal components for all participants in this study. The country codes are based on self-reported ancestry from a subset of 3363 participants. These participants reported four grandparents born in the given country (or in the case of “AJ”, four grandparents of Ashkenazi Jewish ancestry). The label for a country is placed at the median position of all participants reporting such ancestry, and the size is proportional to the number of such reports. Note that the label size is not proportional to the actual density of each subgroup in the study (the densities are approximately 85% northern European and 5% each Ashkenazi, eastern European, and southern European). (TIFF) [file pone.0034442.s001.tif]

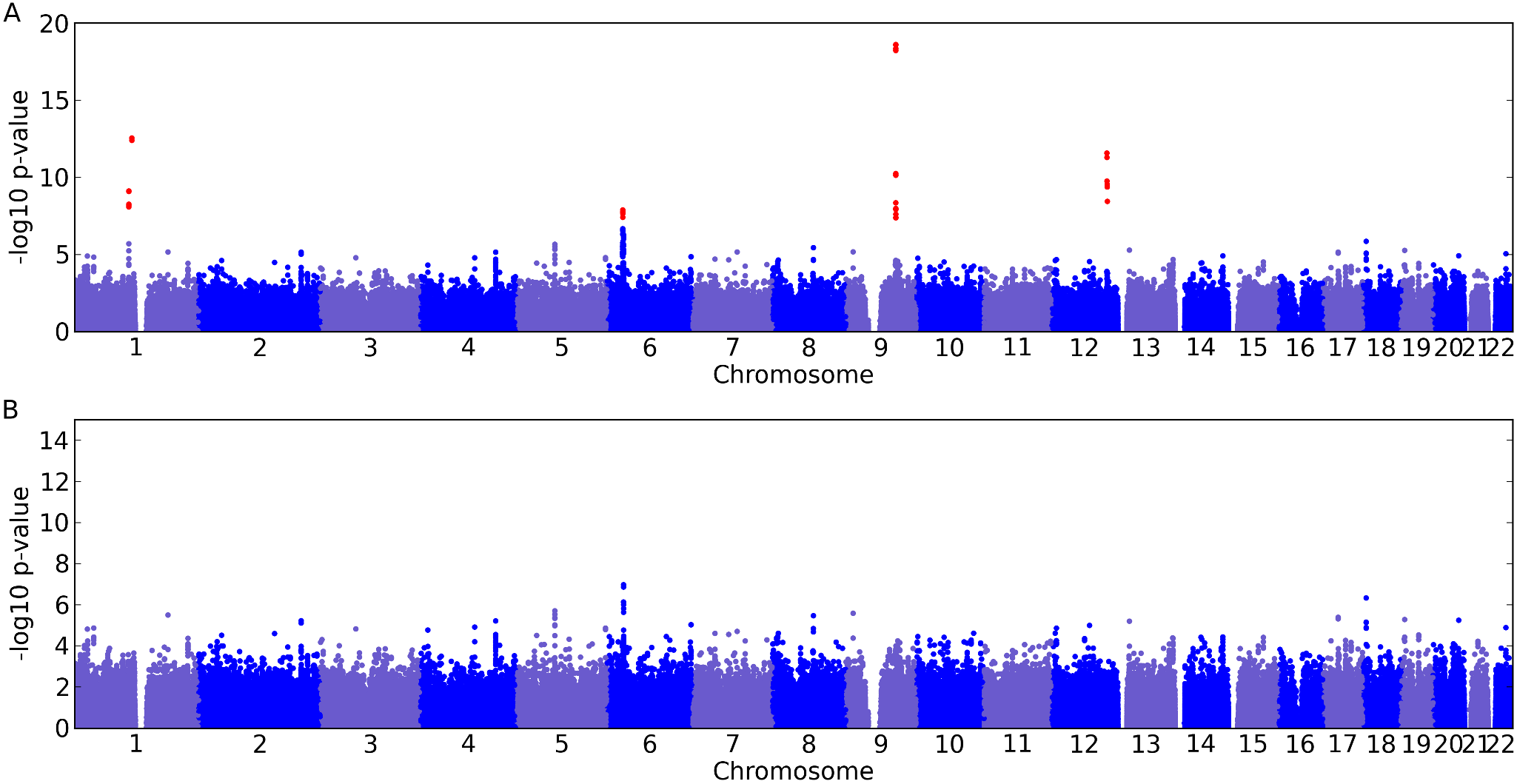

Supplement: Figure S2 — Manhattan plots. (A) Negative log -values for SNPs by genome position. Genome-wide significant SNPs are shown in red. (B) Same for conditional analysis adding 5 genome-wide signficiant SNPs (rs925489, rs6679677, rs3184504, rs4915077, rs2517532) as covariates. (TIFF) [file pone.0034442.s002.tif]

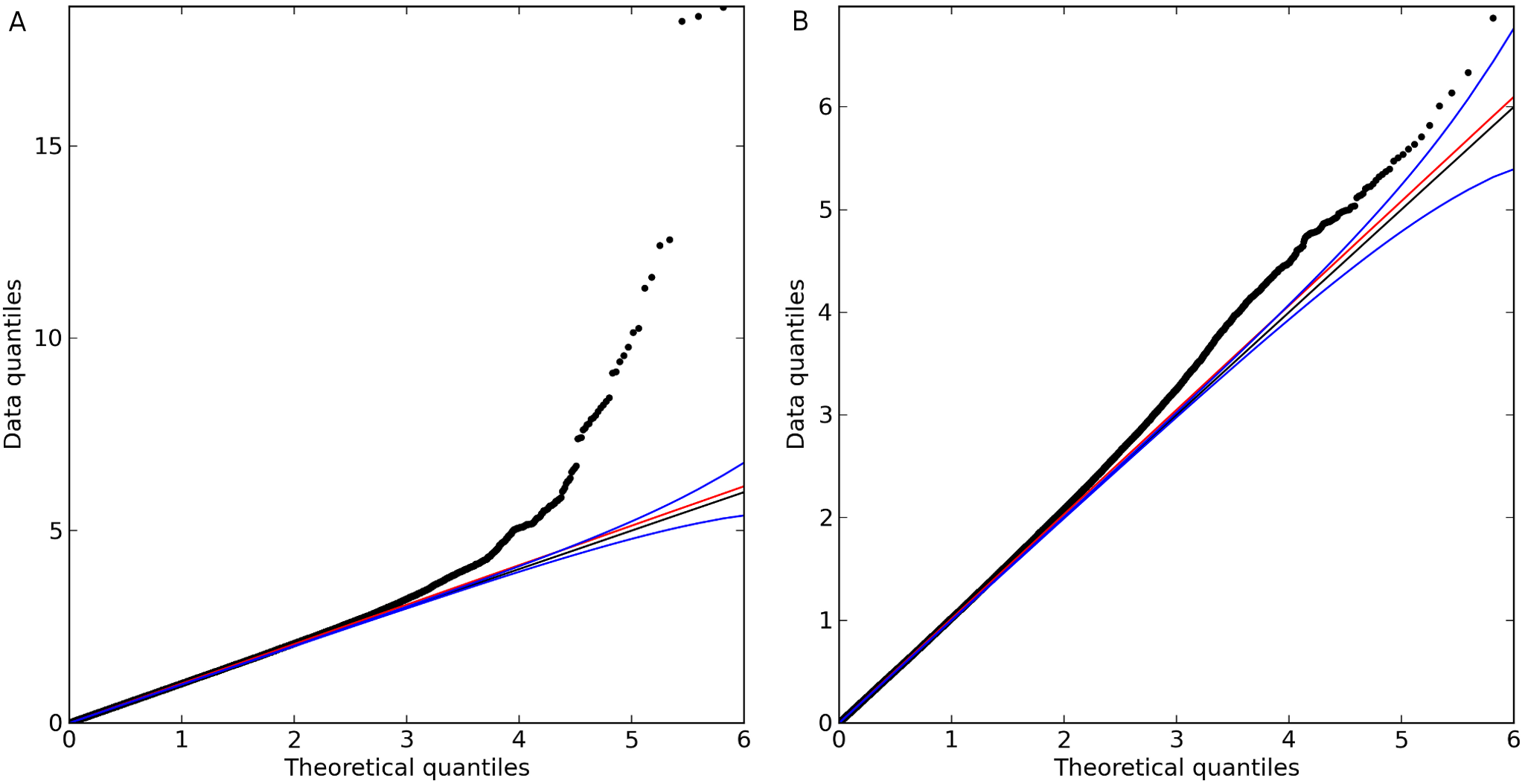

Supplement: Figure S3 — Quantile-quantile plot. Observed -values versus theoretical -values under the null hypothesis of no association. The genomic control inflation factor for the study was and is indicated by the red line. (A) Genome-wide analysis. (B) Conditional analysis with five SNPs included as covariates. (TIFF) [file pone.0034442.s003.tif]

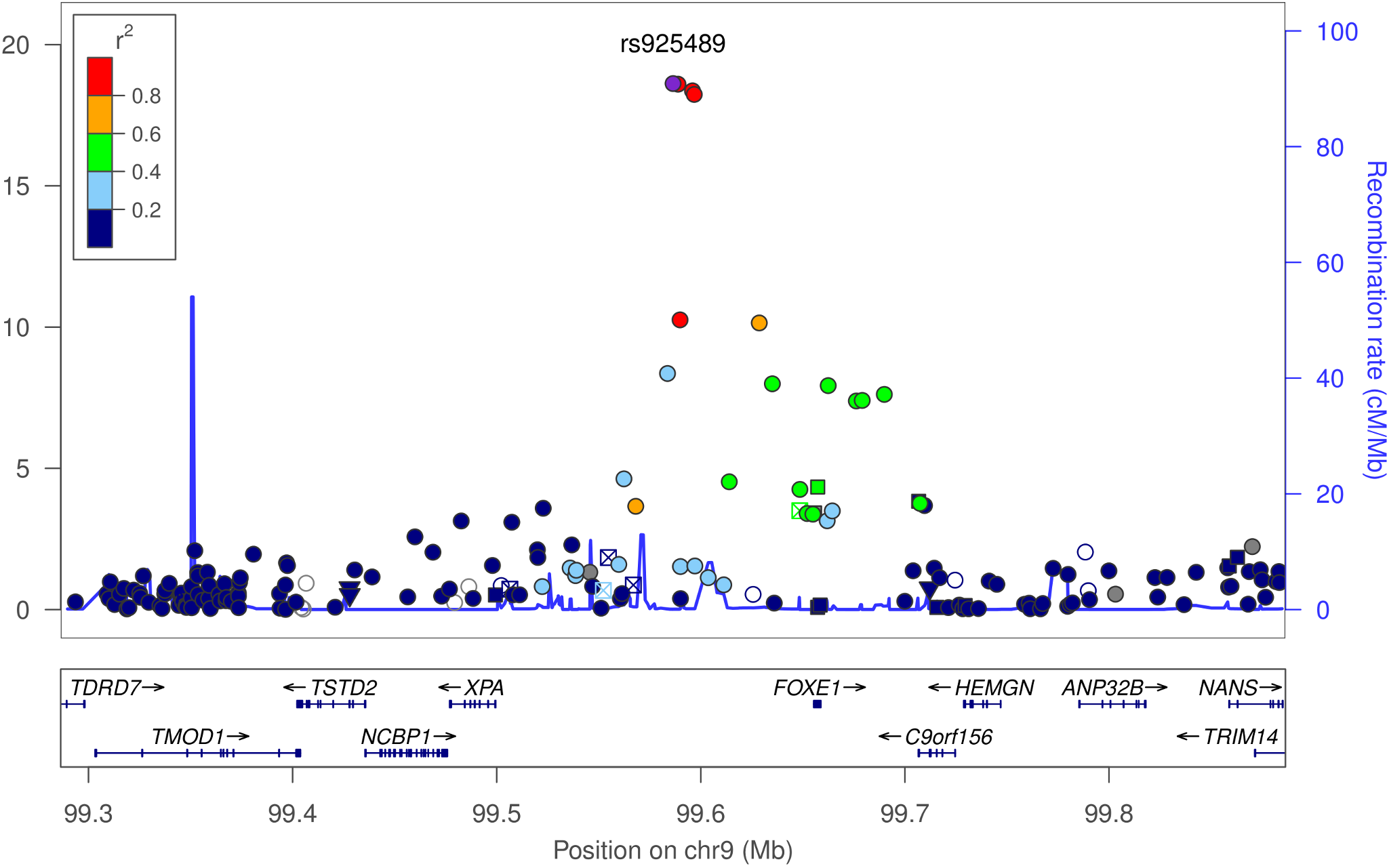

Supplement: Figure S4 — SNPs in the FOXE1 region. In the plot, circles represent unannotated SNPs, upside-down triangles represent non-synonymous variants, and boxes with an “x” are SNPs in regions that are highly conserved across 44 placental mammals. Colors depict the squared correlation () of each SNP with the most associated SNP (i.e., rs925489, shown in purple). Gray indicates SNPs for which information was missing. Plots were produced using the LocusZoom program. (TIFF) [file pone.0034442.s004.tif]

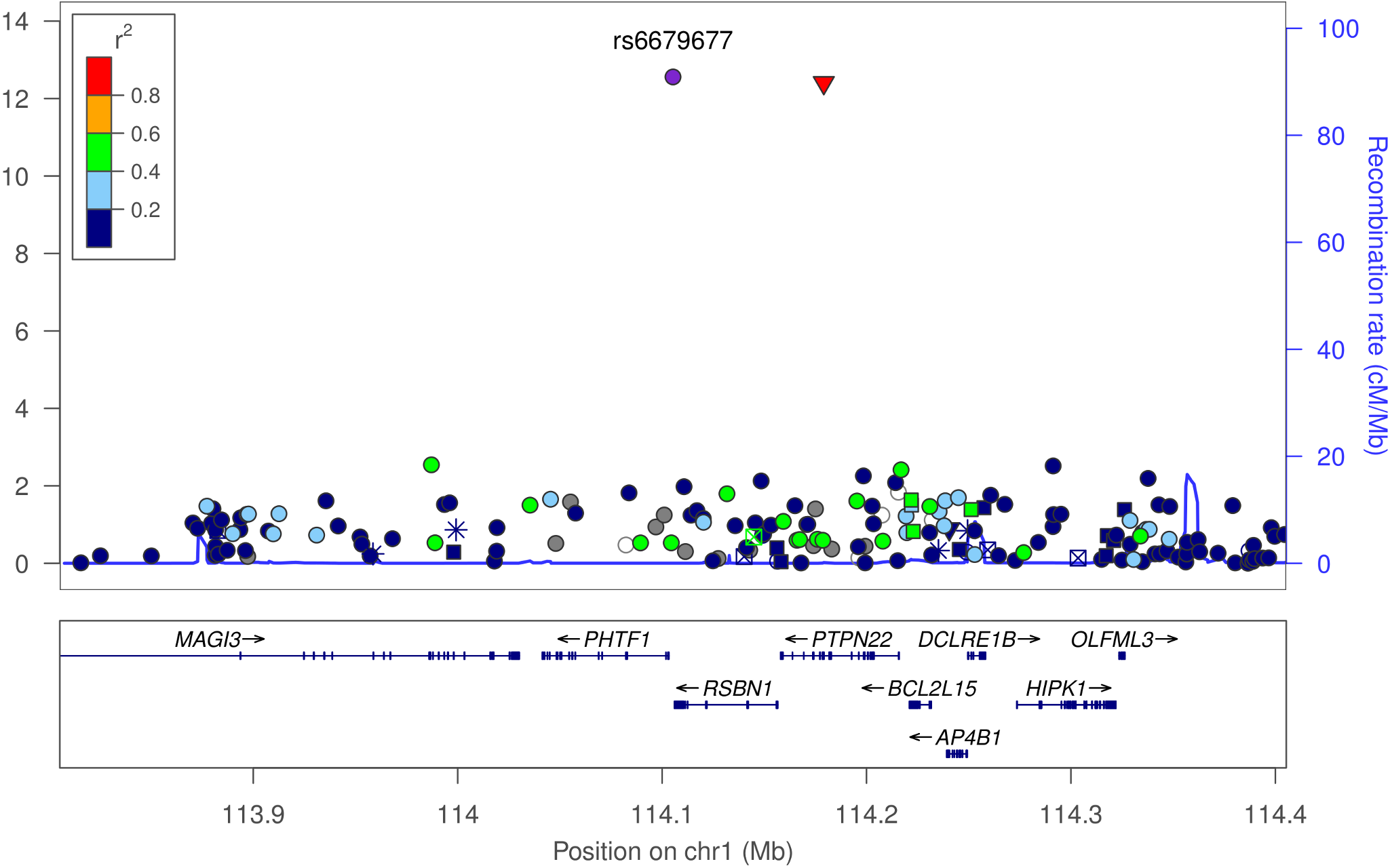

Supplement: Figure S5 — SNPs in the PTPN22 region. For details, see Figure S4. (TIFF) [file pone.0034442.s005.tif]

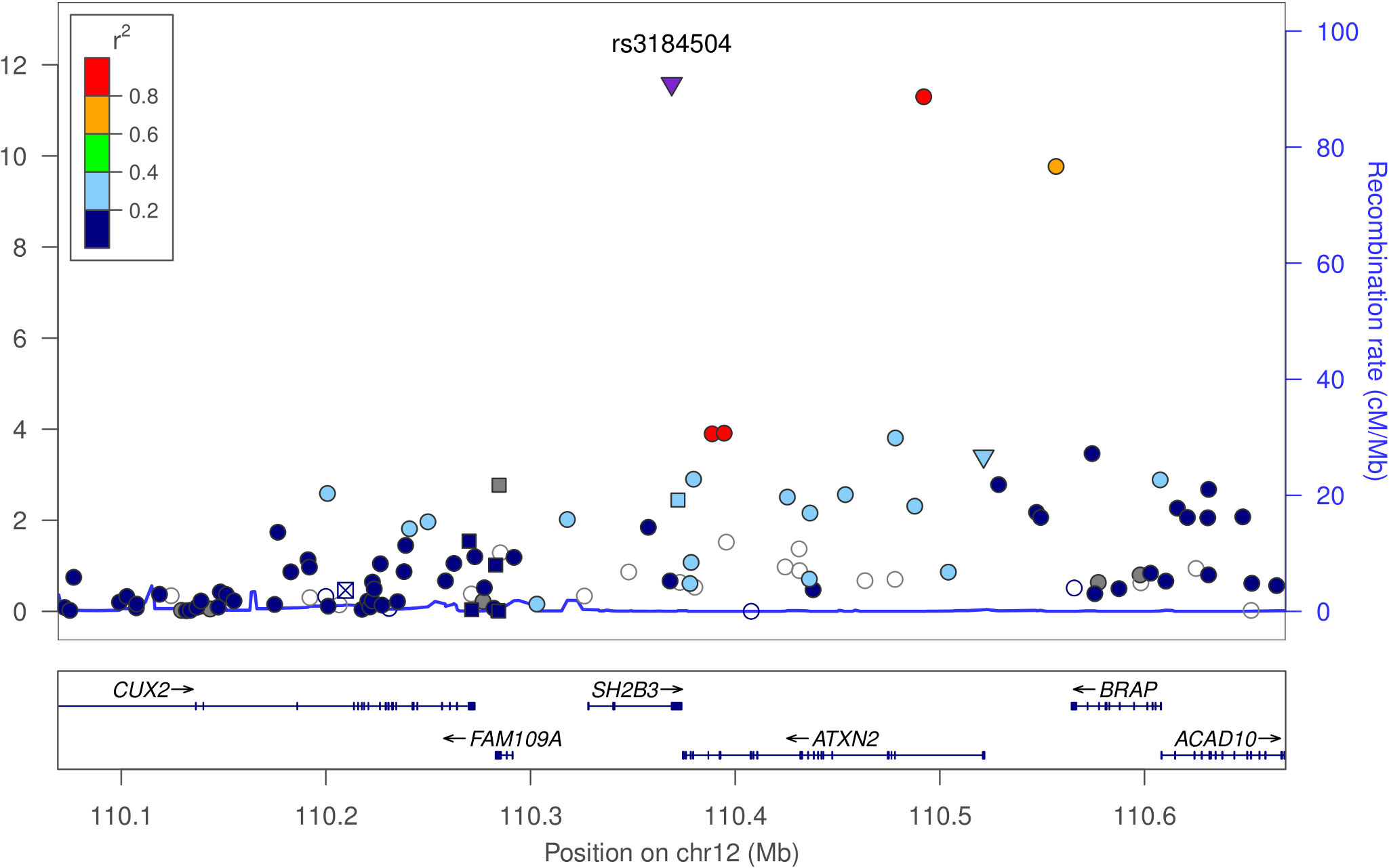

Supplement: Figure S6 — SNPs in the SH2B3 region. For details, see Figure S4. (TIFF) [file pone.0034442.s006.tif]

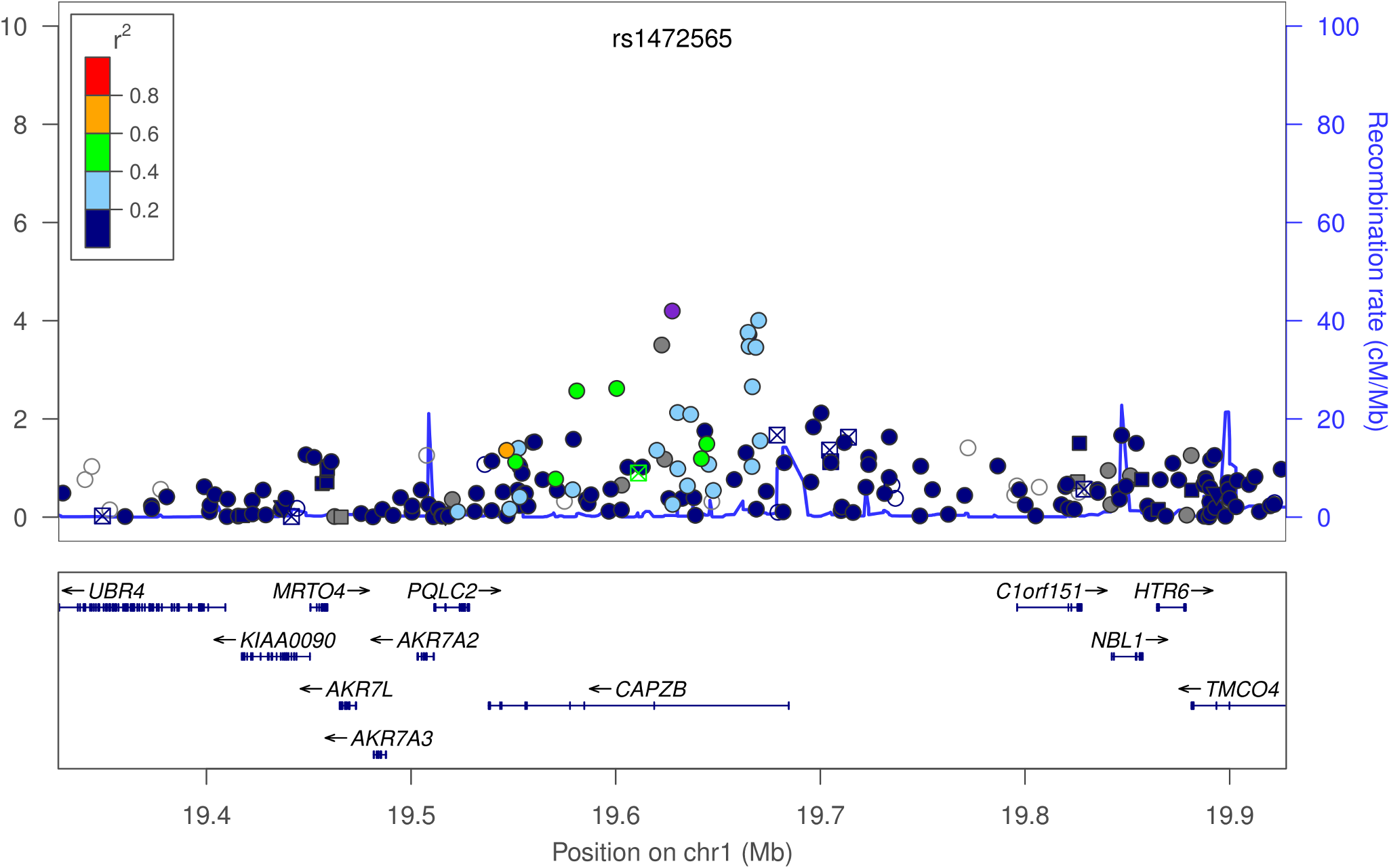

Supplement: Figure S7 — SNPs in the CAPZB region. For details, see Figure S4. (TIFF) [file pone.0034442.s007.tif]

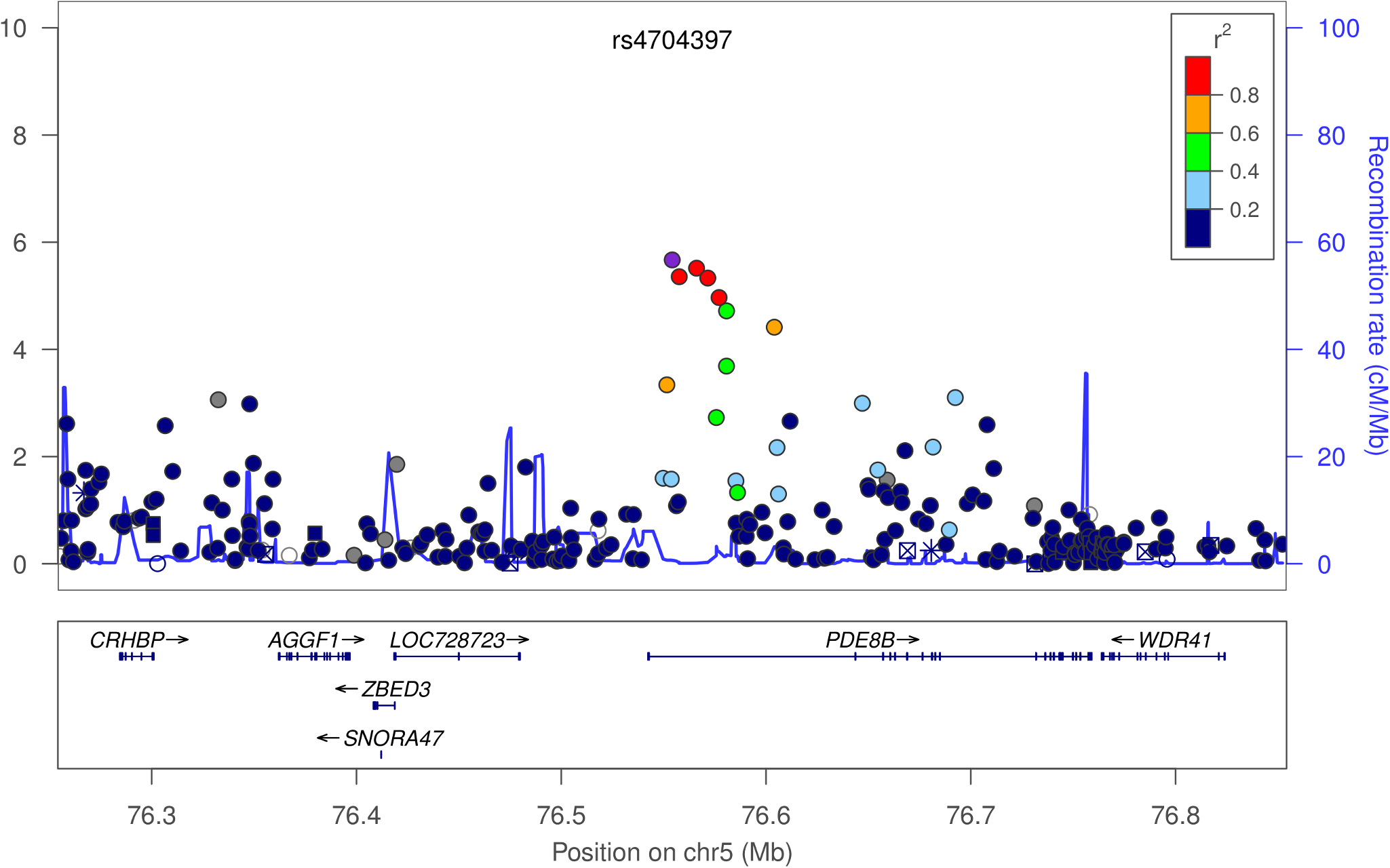

Supplement: Figure S8 — SNPs in the PDE8B region. For details, see Figure S4. (TIFF) [file pone.0034442.s008.tif]

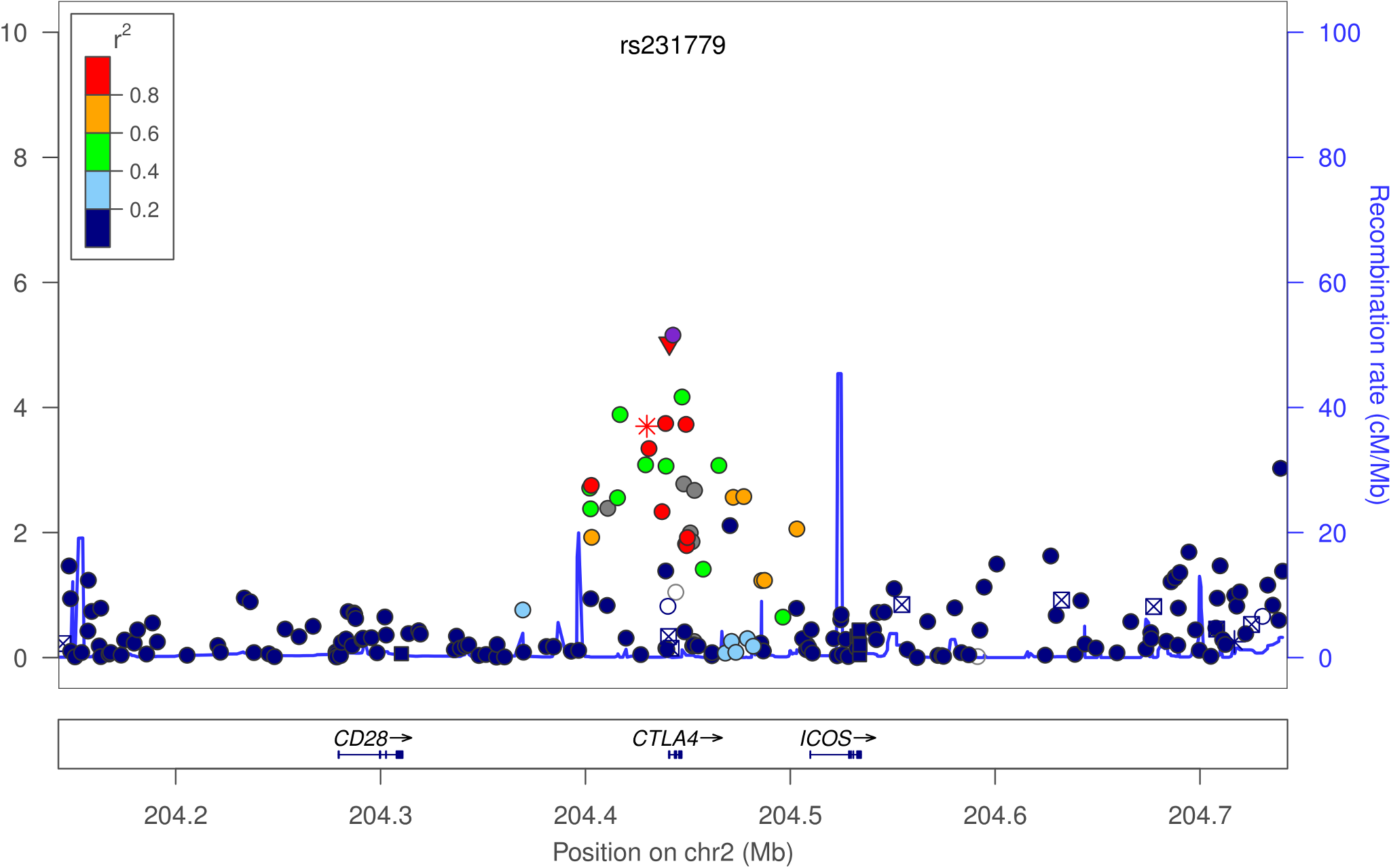

Supplement: Figure S9 — SNPs in the CTLA4 region. For details, see Figure S4. (TIFF) [file pone.0034442.s009.tif]
